# GP perceptions of telehealth services in Australia: a qualitative study

**DOI:** 10.3399/BJGPO.2021.0182

**Published:** 2022-01-26

**Authors:** Keshia R De Guzman, Centaine L Snoswell, Chantelle M Giles, Anthony C Smith, Helen H Haydon

**Affiliations:** 1 Centre for Online Health, The University of Queensland, Brisbane, Australia; 2 Centre for Health Services Research, The University of Queensland, Brisbane, Australia; 3 Faculty of Health and Behavioural Sciences, The University of Queensland, Brisbane, Australia; 4 Faculty of Medicine, The University of Queensland, Brisbane, Australia; 5 Centre for Innovative Medical Technology, University of Southern Denmark, Odense, Denmark

**Keywords:** primary health care, family practice, general practice, telehealth, telemedicine, sustainability

## Abstract

**Background:**

Primary care providers have been rapidly transitioning from in-person to telehealth care during the 2019 coronavirus (COVID-19) pandemic. There is an opportunity for new research in a rapidly evolving area, where evidence for telehealth services in primary care in the Australian setting remains limited.

**Aim:**

To explore general practitioner (GP) perceptions on providing telehealth (telephone and video consultation) services in primary care in Australia.

**Design & setting:**

A qualitative study using semi-structured interviews to gain an understanding of GP perceptions on telehealth use in Australia.

**Method:**

GPs across Australia were purposively sampled. Semi-structured interviews were conducted, recorded, and transcribed verbatim for analysis. Transcripts were analysed using inductive thematic analysis to identify initial codes, which were then organised into themes.

**Results:**

Fourteen GPs were interviewed. Two major themes that described GP perceptions of telehealth were: (1) existence of business and financial pressures in general practice; and (2) providing quality of care in Australia. These two themes interacted with four minor themes*:* (3) consumer-led care; (4) COVID-19 as a driver for telehealth reimbursement and adoption; (5) refining logistical processes; and (6) GP experiences shape telehealth use.

**Conclusion:**

This study found that multiple considerations influenced GP choice of in-person, videoconference, or telephone consultation mode. For telehealth to be used routinely within primary care settings, evidence that supports the delivery of higher quality care to patients through telehealth and sustainable funding models will be required.

## How this fits in

COVID-19 has been a catalyst for increased telehealth uptake in primary care, where high use of telephone compared with videoconference has been observed. Therefore, there is a need to understand the factors that influence GP choice of consultation mode, particularly in the Australian setting. Drawing from semi-structured interviews, this study provides an in-depth understanding of GP perceptions of telehealth, highlights factors that drive GP choice of consultation mode, and identifies unique challenges to telehealth delivery in primary care. Improving the quality of telehealth care, and developing sustainable funding models, will be needed to improve the integration of telehealth services into our existing healthcare systems.

## Introduction

The COVID-19 pandemic has been a catalyst for increased telehealth use globally. Telehealth, the delivery of a healthcare service from a distance, can help prevent potential COVID-19 transmission because it reduces person-to-person contact.^1,2^ As a result, many primary care providers have been rapidly transitioning from in-person to telehealth care.^2–4^ Payers, such as government agencies and health insurers, facilitated this transition by expanding telehealth coverage and reimbursement during COVID-19.^3,5^ This has led to significant increases in telehealth in primary care settings.^6,7^ The rapid uptake and widespread implementation of telehealth since COVID-19 has revealed challenges for incorporating telehealth services in primary care.^7,8^

Primary care is considered the frontline of the healthcare system. In Australia, primary care services are mainly delivered by general practitioners (GPs) and subsidised by the Australian Government through the Medicare Benefits Schedule (MBS). Before COVID-19, MBS telehealth funding was restricted to specialist video consultations for rural or remote patients.^6,9^ This meant that telehealth use by GPs has long been underutilised, mainly because of ineligibility for telehealth reimbursement.^6,7^ However, this changed in March 2020, during COVID-19, when MBS reimbursement was introduced for telephone and video consultations provided by GPs and other clinicians.^6,7^ Compared with Australia, similar telehealth funding changes have been introduced in Canada and the US.^3,5^ This has enabled GPs to continue to provide care directly to their patients, while mitigating the risk of COVID-19 transmission.

In Australian primary care settings, telephone has been predominantly used instead of videoconference.^10^ This has raised many questions regarding the factors that drive GP choice of consultation mode (telephone, videoconference, or in-person). Given the introduction of COVID-19 telehealth reimbursement, there are also questions on the future sustainability of telehealth funding.^11,12^ The natural experiment of telehealth use during COVID-19 has created opportunities to learn from new experiences,^13,14^ and there has been emerging research exploring telehealth use in primary care.^15–18^ As this research progresses, an in-depth understanding of GP perceptions regarding telehealth remains limited in the Australian setting. This study investigated GP perceptions on providing telehealth (telephone and video consultations) services in Australia.

## Method

Semi-structured interviews with GPs provided an in-depth understanding of GP perceptions of telehealth use in primary care. The interview questions explored the rationale behind GP choice of consultation mode (telephone, videoconference, or in-person), telehealth-related experiences during COVID-19, and views on future telehealth use. Demographic questions were also included. Signed, email, or verbal consent (recorded at the start of the interview) was provided by all participants. The results reported aligned with the Consolidated criteria for reporting qualitative studies (COREQ) framework.^19^

### Participant selection and setting

A purposive sample of GPs across Australia were invited to participate in this study. Purposive sampling aimed to recruit GPs with varied telehealth experiences. Recruitment occurred via direct invitation to GPs known to the research team with subsequent snowball sampling, social media, and professional association communications. Recruitment ceased once data saturation was reached.^20^ There was no compensation for study participation.

### Data collection

Interviews occurred via telephone or videoconference and were conducted by author KD, who followed a semi-structured interview guide (Supplementary file 1). Interviews were recorded and transcribed verbatim for analysis. All identifying information from transcripts was removed to maintain participant confidentiality.

### Data analysis

An inductive thematic analysis, as described by Braun and Clarke,^21^ was undertaken to identify initial patterns and codes, which were then organised into themes. Analysis was guided by Lincoln and Guba’s^22,23^ criteria for trustworthiness, which assures that systematic processes have been undertaken throughout analysis in order to remain true to GPs’ accounts. Details were given on how trustworthiness criteria were addressed (Supplementary file 2). Microsoft Excel software was used to organise initial codes and transcripts. Author KD conducted and transcribed the interviews, which allowed immersion in the data,^22,24^ before coding and generating initial themes. A second author (CS) independently generated initial themes from three transcripts (approximately 20%). Three peer-debriefing sessions with authors KD, CS, HH, and AS were conducted, enabling review and finalisation of higher order themes. Author KD explained how reflexivity contributed to the findings (Supplementary file 2).^24^

## Results

Of the fourteen GPs interviewed, the majority were male (64%) and most (57%) had been registered practitioners for >20 years (Table 1). They were aged between 36 years and 73 years (mean [standard deviation {SD}] = 49 [11] years) with most (71%) practising in the state of Queensland, Australia. Six GPs (43%) had spent over half their career time in metropolitan settings. GP participants had varied telehealth experiences, although most consultations per week were delivered in-person. Of the five GPs that delivered more than 25% of consultations per week via telephone, three had spent most of their career time in metropolitan settings, while the other two had spent most of their career time in a regional or rural and remote area. Interviews took place for 20–60 minutes (mean [SD] = 41 [12] minutes).

**Table 1.** Participant demographic characteristics

Characteristic			***n*** (%)
Sex		Male	9 (64)
		Female	5 (36)
Age, years		21–30	0 (0)
		31–50	8 (57)
		>50	6 (43)
Years of registration		1–10	3 (21)
		11–20	3 (21)
		>20	8 (57)
Highest level of training		Bachelor	4 (29)
		Graduate certificate or diploma	2 (14)
		Masters	4 (29)
		PhD	4 (29)
State of current practice		Queensland	10 (71)
		New South Wales	2 (14)
		Victoria	2 (14)
Practice setting	Proportion of career time spent working in metropolitan setting	0 to ≤25	3 (21)
		>25 to ≤50	5 (36)
		>50 to ≤75	1 (7)
		>75	5 (36)
	Proportion of career time spent working in regional setting	0 to ≤25	9 (64)
		>25 to ≤50	4 (29)
		>50 to ≤75	1 (7)
		>75	0 (0)
	Proportion of career time spent working in rural or remote setting	0 to ≤25	10 (71)
		>25 to ≤50	0 (0)
		>50 to ≤75	2 (14)
		>75	2 (14)
Consult modality	Proportion of telephone consultations per week	0 to ≤25	9 (64)
		>25 to ≤50	4 (29)
		>50 to ≤75	0 (0)
		>75	1 (7)
	Proportion of video consultations per week	0 to ≤25	11 (79)
		>25 to ≤50	2 (14)
		>50 to ≤75	1 (7)
		>75	0 (0)
	Proportion of in-person consultations per week	0 to ≤25	2 (14)
		>25 to ≤50	0 (0)
		>50 to ≤75	10 (71)
		>75	2 (14)
Self-rated telehealth experience (from 1 to 10)		1–4	1 (7)
		5–7	5 (36)
		8–10	8 (57)
Out-of-pocket cost charged for non-concessional patients		Yes	8 (57)
		No	6 (43)

### Overall themes

Two major themes described GP perceptions of telehealth services. These were as follows: (1) existence of business and financial pressures in general practice; and (2) providing quality of care in Australia. GP responses highlighted the importance of providing high quality care to patients, which was deeply intertwined with the business and financial needs within general practice settings. These two major themes interacted with four other minor themes: (3) consumer-led care; (4) COVID-19 as a driver for telehealth reimbursement and adoption; (5) refining logistical processes; and (6) GP experiences shape telehealth use.

### Major themes

#### (1) Existence of business and financial pressures in general practice

GPs discussed the existence of business and financial pressures as a major influencer on their choice of consultation mode. They explained that patient care is heavily driven by the financial aspects of private businesses, highlighting how time pressures within busy practices often push them towards shorter consultations. Such pressures often led GPs to favour telephone over videoconference modes. Participants explained that phone consultations are quicker to deliver, enable a higher number of consultations, and yield greater financial benefits compared with videoconference consultations:

*'I don’t do video,* [because] *it takes time. I do telephone.'* (GP6)

*'I think that clinicians have figured out that it’s quick to phone, and fewer patient problems are raised making the length of consultation shorter, and the Medicare rebate is the same for those two services. So, they’re tending to vote with what is more financially appealing.'* (GP19)

However, GPs reported that the busy environment of general practice and the delivery of shorter consultations, irrespective of mode, often leads to rushed care delivery that addresses only straightforward problems raised by patients. Consistently, participants discussed the difficulty of balancing complex patient presentations (that may require longer consultations) with the need to manage workload quickly in a high-paced environment:

*'I had one really experienced GP tell me that when he was running late, he had to pretend to ignore the complexity of the person in front of him.*' (GP40)

*'Limiting it to brief consultations, not longer consultations, not mental health consultations, not chronic disease care planning consultation will end up disadvantaging the patient.*' (GP54)

In these discussions, most GPs expressed regret that this was the reality of the situation, as their preference would be to provide higher quality care, rather than compete with time.

#### (2) Providing quality of care in Australia

GP participants highlighted their desire to provide high quality patient care to ensure optimal patient outcomes. They discussed how the current fee-for-service funding model sometimes disincentivises higher quality care. This tension between quality of care and financial pressures was challenging for GPs, as they described difficulties of providing high quality care in a time-pressured and profit-focused environment:

*'You talk about whole-person care, but how do you do that in 10 minutes? I said, I don’t do it based on Medicare numbers. I do it on what’s good quality of care.'* (GP40)

When comparing telehealth with in-person consultations, GPs had mixed views on quality of care. They highlighted that in-person consultations are needed for physical examinations and added that more subtle aspects of care may be missed though telehealth. However, they also discussed that a physical examination is often not required to provide comparable care:

*'You miss that tactile, therapeutic sort of presence, and you miss the sense of what, that you sort of get face to face.*' (GP71)

*'I reckon this is the truth that a lot of doctors weren’t going to really say, we could still do good quality care 90 to 95% of the time on telehealth.*' (GP11)

GPs further believed that videoconference would provide higher quality of care compared with telephone because it enables visual assessment of patients:

*'The visual interaction, you can see the gestures, you can see the facial expressions, and it’s nicer to see someone. I mean if we’re having this by phone, we couldn’t get the same information. You’re nodding and I’m nodding, and I’m using my hands, there’s more of a conversation that’s occurring.*' (GP80)

Despite the potential for telehealth to provide high quality care, GPs felt that telehealth should complement, rather than replace, the traditional in-person model of care. To improve the quality of telehealth care, they highlighted the need for continued research:

*'There’s potentially a whole field of research that could open up as a result of consultations being done over the phone to make them safer and more effective.*' (GP84)

### Minor themes

#### (3) Consumer-led care: patient experiences, preferences, and needs

Consumer-led care was highlighted as an important factor in influencing GP decisions to provide telehealth. They spoke about how consumer-led care is driven by business needs, such as keeping existing patients or attracting new patients, and described the importance of patient satisfaction to obtain business profits. The role of high quality care was also discussed to create positive patient experiences and promote financial gain:

*'We have to woo clients in. So, it’s a consumer-led thing. I am really big on giving consumers what they want. So, if it said, 60 or 80% of patients want the option of telecare … and if you don't do it, your practice is going to look old and dated.*' (GP11)

*'The patient experience is better, their satisfaction is higher, which leads into the ability to charge, which is, you know in a private business, a big deal … As a GP and practice owner, you’re more likely to feel comfortable billing someone for a video because it’s a rich experience.*' (GP54)

GPs also spoke about the importance of patient preferences and needs, and how choice of consultation mode is often dictated by patients:

*'Phone consultations are driven by patients. They want a phone consultation so they're going to be satisfied with that. They'll not see it as a limit.*' (GP80)

Patient clinical needs as a driver for choice of consultation mode was evident as GPs described how telehealth can provide flexible and convenient care for some patient cohorts (for example, palliative care mobility issues). They expressed how telehealth can meet the care needs for busy people, or people in rural or remote areas. They discussed how access to specialist care is often needed for these patients, with the additional benefit that multidisciplinary video consultations can increase networks between care providers:

*'Palliative care patients, or patients in chronic pain, whereby it’s much more convenient if they’re able to give us a call.*' (GP28)

*'I think that if it can strengthen the existing setups that we have to deliver telehealth between rural and metro, particularly with specialist services, then I think that would be fantastic.*' (GP71)

In further meeting patient needs, GPs described clinical presentations where telephone may be most effective (for example, repeat script requests, provision of results, follow-up consultations, and discreet care for patients experiencing domestic violence). They mentioned videoconference is more effective than telephone for scenarios that require visual assessment, such as skin or throat issues. They emphasised the importance of considering clinical needs when choosing consultation mode:

*'Yeah, and then sometimes you might say, I want you to come back, I want to reassess you clinically.*' (GP55)

#### (4) COVID-19 as a driver for telehealth reimbursement and adoption

GPs discussed how the availability of COVID-19 telehealth reimbursement, introduced to help reduce disease transmission, was the main driver for telehealth adoption. Some expressed that they had delivered telephone consultations before COVID-19 at a cost to themselves. The importance of reimbursement was highlighted when they described how before COVID-19, patients paid for telehealth out-of-pocket, and now these patients were finally able to receive subsidised care:

*'All telehealth started after COVID, after the government funding, I started doing telehealth*.' (GP6)

*'I have used telehealth before COVID came for really remote patients, and often that was not funded by Medicare. They funded it themselves. COVID was a real relief, because those patients had suffered a lot from their remoteness and were now able to get help.*' (GP40)

GPs explained that telehealth reimbursement is important for meeting financial needs of general practice. They raised concerns regarding discontinuation of COVID-19 telehealth funding and expressed the hope for continued telehealth reimbursement. They further emphasised that ongoing funding would be needed for sustainable provision of telehealth services:

*'If the government either stops it or pulls it back, I'll tell you that we will go down kicking and screaming, as will our patients*.' (GP11)

*'The things to capitalise on is, how can you extend beyond the transactional remote care staff to broadening the team, to deepen the care, and to doing it in a sustainable model which is key.*' (GP54)

#### (5) Refining telehealth logistical processes

GPs described multiple logistical issues that arose after rapid telehealth implementation during COVID-19. They highlighted the need to refine logistical processes in order to improve telehealth delivery. They explained how lack of infrastructure, particularly for videoconference consultations, made it challenging to provide telehealth:

*'If we had more infrastructure, then we would be shown how to use it, but we’ve just never had it properly set up. I think there’s only been two cameras in clinic, and so they would be moved around to different rooms.*' (GP28)

To overcome this lack of infrastructure, participants spoke about how they created purpose-built digital platforms or shouldered infrastructure costs themselves (for example, paying for software registrations or buying second mobile phones for business purposes). They further emphasised how improving this infrastructure might enable them to provide higher quality care:

*'If we could improve the quality of the experience, to get it closer to that face to face; I think technology, education, and access to the equipment would bridge that gap.*' (GP71)

Although GPs expressed concerns with how telehealth implementation interrupts administration processes (that is, organising GP referrals, scripts, or consultation payments) and clinical workflows, the need to integrate telehealth services within existing systems was continually emphasised:

*'I think it'll be naturally embedded into everyday practice … we now can flex up and down massively without it changing our workflows because it’s embedded.*' (GP19)

#### (6) GP experiences shaped telehealth use

GPs described how their own experiences shaped their use of telehealth. Some expressed that their colleagues were often resistant to new experiences and most described how the delivery of new telehealth services was an ongoing learning process. They spoke about how they had to adapt their practice for telehealth delivery:

*'Having to use it in different settings, using it more, and then in situations where we probably wouldn't normally use it, we would prefer to get people in to see them. We were having to adapt a bit to use it in that respect.*' (GP28)

GPs spoke about their experiences with building patient rapport through telehealth. Most felt that telehealth may be more appropriate for existing rather than new patient relationships. However, they emphasised that videoconference could help foster more patient rapport compared with telephone because of the visual experience. They described the importance of visual cues to help develop rapport and therapeutic relationships, which are important aspects in providing high quality care:

*'Just from a relationship point of view, I think, having the video would be better than just speaking on the phone.*' (GP84)

*'I think that you're going to find out more patients are going to trust you more, so your advice will be more likely to be understood and acted upon, that’s quality of care.*' (GP80)

They further discussed how telehealth enabled them to increase their connections with patients and increase their follow-up consultations. They felt that telehealth created opportunities for increased patient care through these patient connections. This not only added to relationship building, but also enabled them to achieve greater continuity of care:

*'An urgent result came in, the practice notified me. I could just ring the patient, and I made sure for him to go to hospital. And I just did that, speaking to him for half an hour; it allows us to have our finger on the pulse a lot more.*' (GP11)

## Discussion

### Summary

This study investigated GP perceptions on providing telehealth services in Australia, exploring GP rationale behind choice of consultation mode. Several factors influenced GP decisions to provide telehealth services. Some of these factors included GP desire to provide high quality care, the importance of consumer-led care, and the ability to establish patient rapport. Consideration of these factors was often impacted by the time-pressured and profit-focused environment within general practice settings. GPs hoped that telehealth reimbursement, which was introduced during COVID-19, would continue post-pandemic. This would enable primary care providers to continue providing telehealth services to their patients in the future.

### Strengths and limitations

GPs volunteered to participate in this study; therefore, GPs with strong views (positive or negative) on telehealth may have been more likely to participate because of specific interests in telehealth. Health system structures, funding models, and government policies differ across countries, so these findings may not be applicable outside Australia. The dynamic situation of COVID-19 may have affected participant responses. This is because GP experiences could have evolved while government policy changes were occurring, which may influence consistency of the findings. Information regarding whether GPs were practice owners was not collected and may be useful for future analysis as this may have impacted their responses. Study recruitment of GPs was particularly challenging; this may have been due to time constraints in busy general practice settings. However, exploration of the perceptions of this hard-to-reach population, whose voices are vital to hear, was achieved. The use of qualitative interviews enabled in-depth understanding of GP experiences, which is particularly important as there has been limited investigation into GP telehealth use in Australia. A regular peer-debriefing process enabled enhancement of trustworthiness.^24^

### Comparison with existing literature

An Australian study found that primary care nurses were frustrated with telehealth funding during COVID-19.^25^ Similarly, another study reported that availability of telehealth funding was a barrier to telehealth adoption.^26^ This need for telehealth funding is consistent with the present study, as the need for ongoing telehealth reimbursement was highlighted. Further to this, time pressures and business needs within general practice have been identified in other research as factors that influence GP service delivery.^25,27–29^ In the current study, financial and business pressures were also found to influence GP delivery of telehealth services.

Telehealth implementation in primary care results in novel and flexible ways of working.^27^ A study in the US showed how flexibility, in the context of constant change during COVID-19, was required for telehealth transition.^30^ This supports the current study findings regarding the ways GPs adapted their practice to deliver telehealth during COVID-19. The need to refine logistical processes to improve telehealth delivery has been continuously reported in the literature,^11,25,30–33^ which supports GP discussions on the need for improved infrastructure and streamlined telehealth processes.

The visual element of care, such as non-verbal communication, was found to foster patient rapport and therapeutic relationships. Therefore, videoconferencing may improve patient rapport compared with telephone interactions, resulting in higher quality care provided. One study reported that good patient rapport was a facilitator for telehealth consultations,^31^ supporting that the establishment of patient rapport is needed for effective telehealth delivery. Another study discussed how the ability for clinicians to address non-verbal cues in video consultations would create care that closely resembles in-person consultations.^34^ This further reflects the importance of the visual component of care, which can be achieved through videoconference and not telephone modes.

In-person consultations have been reported as preferable to telehealth consultations for complex clinical presentations.^32^ A study of 25 Australian primary care nurses reported that telehealth does not support complex clinical assessment.^25^ However, this current study suggests that the ability to manage complex presentations is more reliant on consultation length than consultation mode. GPs described how shorter consultations and rushed care delivery hinders the ability to meet complex clinical needs. This is supported by Sturmberg *et al,* ^29^ which discussed how reduced consultation time threatens high quality care.

### Implications for research and practice

There has been investigation into factors that have affected overall telehealth uptake in Australia;^35^ however, specific examination of telehealth use in the primary care sector is lacking. COVID-19 has been a catalyst for widespread implementation of telehealth services in primary care, and there have been many questions raised on the reasons for high use of telephone over videoconference modes. This study builds on the current evidence by identifying unique challenges that exist for telehealth delivery in primary care. This study highlights how tensions between quality of care, and the financial viability of general practice, affects decisions regarding telehealth delivery. GP services are impacted by the complex interdependencies of organisational levels and government policies.^29^ Therefore, ensuring the sustainability of telehealth, while providing some financial benefits, is important for both patients and GPs. This means that appropriate government policies for telehealth funding models are needed. In Australia, the introduction of COVID-19 telehealth funding included MBS reimbursement for telehealth consultations at parity payment to comparable in-person consultations.^6^ In July 2021, GP telephone reimbursement was capped to 20-minute duration consultations.^36^ In December 2021, the Australian Government announced that temporary MBS telehealth items would transition to ongoing arrangements from January 2022.^37^ Although there have been many policy changes surrounding telehealth reimbursement, this study provides further evidence to support the need for ongoing government funding of telehealth services.^38^

An important consideration with regard to telehealth funding is choice of mode between telephone or videoconference consultations. This study found that multiple considerations drive GP choice of consultation mode. These considerations include patient preferences and specific clinical needs, and the importance of providing high quality care. Previous research has suggested higher reimbursement amounts for videoconference than telephone modes to incentivise videoconference uptake.^5^ However, the motivation to use telehealth in primary care is also a function of other variables, in addition to reimbursement. Choice of mode is further impacted by time-pressured environments in general practice as GPs often opt for shorter telephone consultations. Logistical challenges, such as lack of telehealth infrastructure, may further hinder GPs’ ability to provide video consultations. As the debate over different strategies for telehealth reimbursement continues, it will be important to conform to healthcare budget constraints, without compromising quality of care. The ongoing availability of appropriate telehealth funding is not only essential for GPs but also will be valuable for patients who need access to subsided care.

Telehealth is not intended to replace all in-person consultations, however, this study provided evidence that telehealth can meet a range of clinical needs and be equivalent in quality to in-person care. This study builds on the evidence base for scenarios suited to telehealth consultations, which can help expand the understanding of which clinical presentations are well-suited to telehealth care. This includes potential appropriateness of telephone consultations for repeat scripts or follow-up consultations, and use of video consultations for presentations requiring visual assessment. In addition, telehealth involves a change in practice workflows and the complexity of implementation is often underestimated.^39^ Therefore, progress towards effective telehealth delivery will require overcoming logistical challenges and integrating telehealth workflows within existing systems.

GPs emphasised that multidisciplinary video consultations can strengthen the networks between care providers. Therefore, telehealth may help overcome some of the larger challenges to Australia’s healthcare system, such as fragmented services and poor care coordination.^40^ GPs highlighted how telehealth created increased opportunities for patient care, which means that telehealth could expand the capacity of the primary care sector. These benefits, along with the ability for telehealth to meet specific patient needs, should be considered by decisionmakers responsible for future telehealth policies. These findings can help inform future strategies aimed at strengthening the primary care sector. Improving the delivery of higher quality telehealth care compared with in-person care, and ensuring sustainable funding models, will be necessary to successfully embed telehealth services into existing healthcare systems.
